# Perspectives on which health settings geriatricians should staff: a qualitative study of patients, care providers and health administrators

**DOI:** 10.1186/s12877-025-05691-5

**Published:** 2025-01-18

**Authors:** Eric Kai-Chung Wong, Andrea C. Tricco, Wanrudee Isaranuwatchai, David M. J. Naimark, Sharon E. Straus, Joanna E. M. Sale

**Affiliations:** 1 Faculty of Medicine, University of Toronto, Toronto, ON Canada; 2 Institute of Health Policy, Management and Evaluation, Dalla Lana School of Public Health, University of Toronto, Toronto, Canada; 3 Knowledge Translation Program, Li Ka Shing Knowledge Institute, St. Michael’s Hospital, Toronto, ON Canada; 4 Epidemiology Division, Dalla Lana School of Public Health, University of Toronto, Toronto, Canada; 5 Health Intervention and Technology Assessment Program, Ministry of Public Health, Nonthaburi, Thailand; 6 Sunnybrook Health Sciences Centre, 1929 Bayview Avenue, Toronto, ON M4G 3E8 Canada; 7 Musculoskeletal Health and Outcomes Research, Li Ka Shing Knowledge Institute, St. Michael’s Hospital, Unity Health Toronto, 30 Bond Street, Toronto, ON M5B 1W8 Canada; 8 Department of Surgery, Faculty of Medicine, University of Toronto, Toronto, ON Canada; 9 St. Michael’s Hospital, Unity Health Toronto, 30 Bond Street 1st Floor Bond Wing, Room 1-420, Toronto, ON M5B 1W8 Canada

**Keywords:** Geriatrician, Qualitative study, Comprehensive geriatric assessment, Healthcare setting, Staffing, Implementation science

## Abstract

**Background:**

With a shortage of geriatricians and an aging population, strategies are needed to optimise the distribution of geriatricians across different healthcare settings (acute care, rehabilitation and community clinics). The perspectives of knowledge users on staffing geriatricians in different healthcare settings are unknown. We aimed to understand the acceptability and feasibility (including barriers and facilitators) of implementing a geriatrician-led comprehensive geriatric assessment (CGA) in acute care, rehabilitation, and community clinic settings.

**Methods:**

A qualitative description approach was used to explore the experience of those implementing (administrative staff), providing (healthcare providers), and receiving (patients/family caregivers) a geriatrician-led CGA in acute care, rehabilitation and community settings. Semi-structured interviews were conducted in Toronto, Canada. The theoretical domains framework and consolidated framework for implementation research informed the interview guide development. Analysis was conducted using a thematic approach.

**Results:**

Of the 27 participants (8 patients/caregivers, 9 physicians, 10 administrators), the mean age was 53 years and 14 participants (52%) identified as a woman (13 [48%] identified as a man). CGAs were generally perceived as acceptable but there was a divergence in opinion about which healthcare setting was most important for geriatricians to staff. Acute care was reported to be most important by some because no other care provider has the intersection of acute medicine skills with geriatric training. Others reported that community clinics were most important to manage geriatric syndromes before hospitalization was necessary. The rehabilitation setting appeared to be viewed as important but as a secondary setting. Facilitators to implementing a geriatrician-led CGA included (i) a multidisciplinary team, (ii) better integration with primary care, (iii) a good electronic patient record system, and (iv) innovative ways to identify patients most in need of a CGA. Barriers to implementing a geriatrician-led CGA included (i) lack of resources or administrative support, (ii) limited team building, and (iii) consultative model where recommendations were made but not implemented.

**Conclusions:**

Overall, participants found CGAs acceptable yet had different preferences of which setting to prioritise staffing if there was a shortage of geriatricians. The main barriers to implementing the geriatrician-led CGA related to lack of resources.

**Clinical trial number:**

Not applicable.

**Supplementary Information:**

The online version contains supplementary material available at 10.1186/s12877-025-05691-5.

## Background

The comprehensive geriatric assessment (CGA) is a multidimensional approach to assessing and managing older patients with multimorbidity and frailty [1]. Randomized trials have demonstrated the effectiveness of CGA in decreasing long-term care (LTC) admissions [2–4], functional decline [2, 3], hospital readmissions [5], health resource utilization [6], and death [2, 3] in various healthcare settings. The CGA can be led by a geriatrician or by other health providers, and preliminary data from our research group showed that a geriatrician-led CGA has efficacy benefits over other health providers [7]. Geriatricians receive specialized training in both internal and geriatric medicine and have deep understanding of various diseases in older adults, but the number of geriatricians in Canada are limited (0.57 full-time equivalents for every 10,000 people aged ≥ 65 years in 2019) [8]. Through an economic evaluation (under review) [9], we found that staffing geriatricians in acute care and rehabilitation hospitals is the most cost-effective strategy if resources are limited (e.g., limited number of geriatricians or limited healthcare funding). If more geriatricians can be trained, then a combined strategy of acute care, rehabilitation and community clinics is also cost-effective. This knowledge is timely and important for the care of our aging population, with 6.4 million Canadians aged ≥ 65 years estimated to increase to 10 million by 2036 [10]. Canada has a publicly funded, single-payer healthcare system where primary care and hospital care (acute and rehabilitation) are largely covered by the government [11]. Geriatricians are generally remunerated using a fee-for-service model, but some receive extra funding (e.g., academic funding, sessional fees) [12].

Integrated knowledge translation is a collaborative approach that engages knowledge users as equal members of the research team throughout the entire research process from developing research questions to completing studies and implementing research findings [13]. Knowledge users may include patients, caregivers, clinicians and policy makers who use the research evidence to implement change [14]. No study to date has investigated the experiences of various knowledge users in implementing a CGA. A few qualitative studies have examined the patient experience only [15–17] from European countries (e.g., United Kingdom [16], Sweden [17], Netherlands [15]). These studies did not address the quality of care perceived by other knowledge users. Some participants did not recall receiving a CGA [15, 16], which limited the findings available from studies. For participants that recalled the CGA, they reported that being respected as a person [17], having a holistic approach [15] and facilitating functional dependence [16] were key benefits of a CGA. No published qualitative study has examined the acceptability and feasibility (including barriers and facilitators) of implementing a geriatrician-led CGA in different healthcare settings, which is a critical step in the knowledge translation process [18].

The objective of this study was to understand the knowledge users’ perceived acceptability and feasibility (including barriers and facilitators) of the geriatrician-led CGA in acute care, rehabilitation and community clinics to optimise uptake.

## Methods

### Study design, eligibility criteria and recruitment

A qualitative description approach [19] was used to explore the experience of those implementing (administrative staff), providing (healthcare providers), and receiving (patients/family caregivers) a geriatrician-led CGA in acute care, rehabilitation, and community settings. A qualitative description approach was chosen to provide a rich and direct [20] description of the participants’ experiences to inform policy [19]. Reporting of this study conformed to the reflexive thematic analysis reporting guidelines (RTARG) and sex and gender equity in research (SAGER) guidelines [21, 22]. Our analysis adhered to relevant items (i.e., those that apply to qualitative research such as reflexivity) in strengthening the integration of intersectionality theory in health inequality analysis (SIITHIA) checklist [23].

Semi-structured interviews were conducted with patients, care partners, referring physicians, geriatricians, and healthcare administrators. Patients older than 65 years and their family caregivers (interviewed separately) were recruited from St. Michael’s Hospital, Toronto, Ontario, Canada, which is fully affiliated with the University of Toronto. Patients with mild cognitive impairment or mild dementia (as documented on chart by clinical dementia rating scale [24]) were eligible for inclusion with consent from their care partners (e.g., family or substitute decision maker). Verbal consent was obtained prior to interviews. The interviews were conducted from December 2023 to March 2024.

Patients and their care partners were approached by a geriatrician or clinic nurse within their circle of care for permission to be contacted by the research team. Primary care physicians were recruited by looking at the referral sources from a list of referral letters collected from the patient charts by a clinic nurse. Acute care physicians (medical and surgical specialist physicians) were recruited by looking at inpatient referrals. Geriatricians working in three settings (acute care, community clinics, and rehabilitation) were recruited by email. Healthcare administrators managing geriatric services were recruited by email.

The recruitment process aimed to include a diverse group reflective of the Greater Toronto Area population [25] using equity characteristics such as age, gender, sex, language, ethnicity, education, and place of residence [26]. Maximum variation sampling based on the demographic and equity characteristics was used to recruit participants [27, 28]. To promote inclusivity, recruitment was carried out using flexible dates and times (weekends or after hours), a variety of mediums for the interview (video, telephone or in person), and the option of providing an interpreter, and ensuring all materials were written material at grade 7 level [29]. Caregivers were interviewed separately to understand their perspectives. Research ethics board approval was obtained from Unity Health Toronto (23–140) and University of Toronto (45396). The study was conducted in accordance with Declaration of Helsinki. We provided a C$25 gift card to each participant as a token of appreciation.

### Interview guide

The interview guide (Additional file: Appendix 1) was informed by the theoretical domains framework [30], which identifies influences of individual behaviour change. The framework was further adapted to address intersectionality questions, which were employed in this study, as appropriate [31]. Intersectionality refers to the interface between social identity (e.g. age) and structures of power (e.g. ageism) [32]. The 14 domains included the skills, beliefs, roles/identities and social influences of a geriatrician-led CGA. Participants were asked about their experiences with the geriatrician CGA, the value of a CGA, the preferred healthcare setting for geriatricians to staff, the resources required for a CGA, and out-of-pocket costs to patients and caregivers from CGA recommendations. Barriers and facilitators to implementing the geriatrician-led CGA were also explored.

We also used the consolidated framework for implementation research (CFIR) 2.0 to guide our questions [33] because we included administrators and wanted to explore organisational contextual factors. The CFIR is a determinant framework used to explore barriers and facilitators to implementing new health interventions at an organisation level. The interview guide was piloted on three participants (one in each category of patients and their carers, physicians and administrators). Interviews were conducted by a single interviewer (EW) in English either in person, by video conference or over phone.

### Analysis

Recorded interviews were transcribed verbatim and imported into NVivo. Automated transcription was done using Zoom for Healthcare [34] or NVivo transcription [35], and an investigator (EW) reviewed each transcript for errors. Two reviewers (EW and JS) independently coded the first three transcripts to develop a coding template. The rest of the transcripts were coded independently by one reviewer (EW) using the template. The analysis was done inductively using a thematic approach [36]. As described by Braun and Clarke [37], the inductive thematic approach begins with an exploration of the whole dataset and generation of initial codes. Themes were developed around codes that pertained to the research question and were later refined. We analysed barriers and facilitators separately from the themes as they were more consistent with topic summaries as discussed by Braun and Clarke [38]. Our analytic approach was to use the theoretical frameworks only to inform the interview guide but not as an analytic tool [38]. We wanted to generate themes that were relevant to the overall research objective and not confined to domains in the framework [21, 39]. Sex and gender differences were explored in the development of themes [40].

We promoted rigour by selecting an appropriate method for the research question, clearly describing sampling and analysis processes, and supporting claims with direct quotations [41]. Reflexivity was practiced through journaling (EW) and discussion among investigators (JS and EW) [42]. The interviewer (EW) is a geriatrician and PhD student supervised by a geriatrician clinician scientist (SES) and a qualitative methodologist (JS). The interviewer, having conducted an economic analysis (under review) [9], was aware of the research findings of which healthcare setting was most cost-effective. While the interviewer did not provide care for patients in this study, he provided care for patients and caregivers who were not participants in this study. The interviewer attempted to not let these factors unduly influence the interview process and analysis.

## Results

### Participant characteristics

Of the 64 people invited, 27 agreed to participate (Table 1), including five patients (19%), three caregivers (11%), three geriatricians non-administrators (11%), ten health administrators (eight geriatricians and two non-geriatricians, 37%), and six referring physicians (22%). The mean age of the participants was 53 years, and 14 participants (52%) were female. Gender was identified as woman in 14 participants (52%) and man in 13 participants (48%). Nearly all participants had postsecondary education (*n* = 26, 96%). Race was identified as white in 18 participants (67%) and east Asian in six participants (22%). For employment and marital status, 21 participants (78%) were working full-time for pay and 19 participants (70%) were married.

**Table 1:** Participant characteristics

Characteristic	Total n = 27
Participant type, n (%)	
Patients	5 (19)
Caregivers	3 (11)
Geriatricians	3 (11)
Referring physicians	6 (22)
Administrators	10 (37)
Age, mean (SD)	53 (18)
Sex and gender, n (%)	
Female sex	14 (52)
Male sex	13 (48)
Woman gender	14 (52)
Man gender	13 (48)
Education, n (%)	
Postsecondary education	26 (96)
Marital status, n (%)	
Married	19 (70)
Race, n (%)	
White	18 (67)
East Asian	6 (22)
Employment status, n (%)	
Working full-time for pay	21 (78)

### Overview of themes

Participants described a broken healthcare system for older adults (theme 1). Long wait times to see a geriatrician were reported to be an issue by patients, physicians and administrators. Wait times were perceived as demoralizing to geriatricians who reported feeling helpless in an under-resourced health system. Cost saving was viewed as the most important factor driving hospital management decision making. Perceived low prioritisation of geriatric services by hospitals and government policies was attributed to ageism in society. Participants also had varied perceptions of the impact of a geriatrician-led CGA for older adults (theme 2). Some participants shared experiences of others being hesitant to refer for, or to accept, a CGA due to a misunderstanding of its benefits, while others reported strong hospital support for geriatric services when beneficial outcomes were demonstrated (such as decreased length of stay or cost savings).

There were divergent opinions about which healthcare setting was most important for geriatricians to staff (theme 3). Although the geriatrician-led CGA was reported to be beneficial and acceptable in all explored settings, acute care was reported to be most important by some because no other provider had the intersection of acute medicine skills with geriatric training. Others felt that community clinics were most important to manage geriatric syndromes before hospitalization was necessary. The rehabilitation setting was seen as important as well but as a secondary setting. A list of example quotations from themes and subthemes are shown in Table 2.

**Table 2:** Key findings with example quotes. The participant number is followed by a letter, which indicates the participant type (P = patient, C = caregiver, G = geriatrician, R = referring physician, and M = health manager).

Subthemes	Example quotes
**Theme 1: Challenges in navigating the healthcare system for older adults**	
System navigation and wait times	“No obstacles [in getting a CGA], but just in terms of getting… you know, an appointment took a long time… The whole process of getting [wife’s name] diagnosed it took maybe two years… all together two years.” – 13P
Shortage of geriatricians	“I think that’s a massive problem… I think that geriatricians are the best doctors for the patient population that we have now, and that we’ll have for the next 30 years. I really do feel like we’re the only ones who have the training to put together these comprehensive plans for older adults… I’m a big believer in the value of geriatricians in the next 20 to 30 years as… gatekeepers to health resources… I think that that is very bad and terrifying that the match rate is so low.” – 04M
Demoralization of health care providers	“So you know, specialists are demoralized, because they realize they cannot see their patients in a timely way. Like, you know, our waitlist in geriatric medicine is ridiculous. Like we realize it’s foolhardy. You know, someone who’s, you know, [got] an eight month waiting list or whatever it is, you realize that you’re not seeing them, they may not live eight months. So it’s ridiculous, but we do it because we don’t have a way to solve it right now. So like I said, that’s why I think we need real disruptive change. And I’m hopeful that it will happen. It won’t happen from politicians, it’ll happen from us.” – 19M
Big G little g	“I think one of the ways is when all physicians are getting training, whether it’s in hospitalist medicine or internal medicine or surgery, they should all go through a robust geriatric rotation. Because once they are on a consulting geriatric rotation, and they see what our benefits of our services, what we can do, and how we see things from our lens, then those, when they become clinicians in the future, they will remember their training, how geriatrics was very helpful.” – 02G
Cost as the common denominator in hospital decision making	“How do you motivate hospitals? Like it all comes down to money, right? It’s actually not about like patient care. It’s really about money.” – 04 M “If there’s donations targeted to seniors’ care, that would certainly incentivize the hospital more. I think ultimately just the general public expressing more of an interest in seniors’ care, which is unfortunately, I think something that again is not a very shiny area for people to focus on. So, you know, you do see a lot of interest in, for example, fundraising or donations for paediatric care, but seniors’ care tends to be a little bit in the shadows.” – 09M
Outcomes that hospital managers want to see	“And a lot of our system is focused on, you know, again, reaction as opposed to prevention. Partly because preventative medicine, obviously there’s a long time for us to actually see the impact. So it’s like you invest now and you see the benefit in 10 or 15 years, which for governments and institutions might be too long a frame for leaders and executives.” – 20M
Older adults as a priority for hospitals	“There’s a lack of appreciation for what we can offer the geriatric patients that our hospital serves. I think a lot of lip service is paid to being geriatric/senior friendly, that kind of thing. But when I start asking things like, hey, I’d really like to run another clinic on Thursday afternoons, I can hire a new geriatrician, but I would need nursing support, I need a room, I need a room potentially for a resident, a learner with them. Suddenly, it falls on deaf ears. So I guess that has been an ongoing frustration the whole time I’ve been division head, that we’re just not resourced appropriately.” – 06M “It’s just the noise, the hustle, the bustle and everything rather than just going and sitting in a waiting room in a doctor’s office. And it’s a friendlier atmosphere rather than feeling like you’re being passed by. You don’t know why you’re at the hospital.” – 18C “You don’t want it to be ducked away at the way the last floor of the building… like I’m going to send you into a funeral parlour. It should look like the rest of the of the hospital, it should not be a downgrade.” – 22P
Countering ageism in society at large	“I mean, at one time, elders were honoured. They were important. Society, a lot of societies, they were very important. Now, they just become old and useless.” – 21P
**Theme 2: Varied perceptions of the impact of a CGA**	
Benefits of a CGA	“The benefits of seeing everybody with frailty in the hospital… I honestly think hospital stay would be decreased tenfold. I think you’d see bounce back rates to go down by at least 20 to 30% for these patients. I think patients would have incredible medical literacy, at least as they left hospital in terms of how to… really take hold of their own health and wellbeing. It would enable us to properly provide safer doses of chemotherapy… I also think it would be a blessing for family physicians that often get the brunt of discharge summaries that are often lackluster from surgical services.” – 10R “[The geriatrician’s] bedside manner and the way she talked to me and she could tell that I might not be understanding what she’s saying and she would say, ‘okay, let me take a minute and explain that to you’… I think she did more than listen. I think she listened with her body if that makes any sense to you…. I’d also add to that that I think it’s important that everybody experiences what I experienced with that [geriatrician].” – 16P
Perceived consequences of a CGA	“I see geriatric notes that are five and six and seven pages, and I’m astounded… I think the geriatricians know my patients better than anyone, right? But I would have to ask my geriatrician colleague, do they ever get a sense that perfection is the enemy of the good? I’m sensitive to the importance of probabilistic thinking or Bayesian reasoning in our practice of medicine, right? We approach certainty asymptotically, right? Never to arrive, right? But we don’t ever get there. But each step closer and closer towards diagnostic certainty, right, comes with greater expense, greater time, and oftentimes greater risk, right? And I think individuals probably have a different comfort level for how much uncertainty they’re willing to live with in the practice of medicine.” – 03R “From a surgeon perspective, um, not all surgeons want all of their patients to be seen by med consults or geriatrics, for example… cause I think they probably worry that it lengthens their hospital stay. I’m just being honest.” – 12R
Awareness of evidence for a CGA	“The benefits of the CGA are probably not very widely known. So, from a political point of view, for sure it’s not well known. But even in the medical community, probably honestly, if we really put it, including myself, probably even among geriatricians who provide CGAs, we probably like… if I was to sit down and I had to list out the benefits or if you gave me a list of like 50 potential benefits that are evidence-based and maybe 20 of them are true. I probably wouldn’t be a 100% correct. I don’t even know myself… I know a lot of family docs don’t even refer to geriatrics because they don’t really know what the point is because, you know, the opinion is sometimes, well, you can’t treat it.” – 07 M “Well, I mean, you could do it through, I mean, you know, telling regular doctors that this is a service. I mean, does everybody, every GP know this service exists?” – 23P
Out of pocket payments	“$75 for a workbook… [Learning] the ropes memory book. And…transportation, you know TTC [public transit in Toronto] that’s it.” – 13P “I’m driving down, paying for parking, driving my dad back. So really the cost of it for him, he takes the taxi or the TTC [public transit in Toronto], um, and then transportation. And for me, it would be just like my time, which, you know, time off work.” – 15C
**Theme 3: Divergent views on healthcare setting for CGA**	
Acute care as priority	“To me it would be quite clear that it should be absolutely in the hospital so acute care. Outpatient clinics, I think the other roles from my impression could be completed by somebody else who gets extra training like a family doctor or maybe a nurse practitioner. But in the hospital, there is a degree of complexity that requires a geriatrician for sure.” – 05R “I think I like inpatient work more than outpatient, which has come and gone throughout my career, but I’m kind of like more of an inpatient phase right now. And I’ve had arguments with my colleagues about this because some of them feel very strongly we should just be an outpatient-based specialty. But I don’t know, my gut instinct is I think we can do the most. I really do think we can do the most as an inpatient consultation service.” – 06M “From my perspective, inpatient… because as a surgeon, that’s where I see most of my patients. Very little is done of impact in the outpatient setting that doesn’t also centre around an inpatient admission. So I think for me, that’s very important to have that support to really get patients in and out as safely and quickly as possible through their surgical admissions.” – 08R
Community clinic as priority	“I think maybe the outpatient setting to be honest. I think if I look at the work I do in acute care a lot of it is just good internal medicine and an understanding of geriatric syndromes, which I think those that have an affinity for care of older adults can probably do a pretty decent job in collaboration let’s say with a clinical nurse specialist who has additional training in older adult care and services that are available around the city. I think often in the acute care setting the comprehensiveness of our consultation notes are probably not as good as those in the outpatient setting where we have dedicated time to speak with family members, the patient themselves when they’re well and is able to communicate to us kind of their value goals and symptoms.” – 11G “I actually think that the greatest value in terms of geriatric specialty knowledge and comprehensive geriatric assessment is probably in a setting that’s community-based and ambulatory, where you’re actually working on more preventative medicine.” – 20M
Rehabilitation as an option	“But I would say that at least in our environment here, there’s a huge need to support the community and older adults living at home. And so, when you work in a rehab hospital like ours, where you’re supporting things like a falls clinic or outpatient program, which supports family doctors, and then our inpatient setting, which also supports transition back to the community. And we also admit patients directly from the community. You’re also supporting older adults to live at home. And I think that’s a huge need at this time, where we don’t have enough long-term care beds, we don’t have enough often home care support. So, helping the community and older adults stay out of hospital, I think, is a really important part of a geriatrician’s role. Not to say you’re not needed in acute care, but if we can keep older adults out of hospital, then you won’t have to see them in acute care.” – 14M “It’s just because we have a pretty supportive system already. You know, I love having the geriatricians, and they add a lot. But if I had to, if I were sitting as king of the healthcare system, I would direct them to other places instead. My selfish interest is to have them in rehab for my patients. But if I, you know, if I take that away, then I think they have a really big impact in these other locations. Right. So, a greater impact if they’re not provided at those other two settings.” – 26M
Work exposure to all settings	“I do [like to work in all of the settings]. And that’s always been a personal preference, because I feel like we can’t be out of touch with what happens. Right? Like, you know, the reason why they don’t know what happens, like internists have, I’m not trying to like, you know, you know, like bash my internal medicine colleagues, but basically, because, you know, they haven’t done a lot of the outpatient stuff. And they don’t know really what happens. They don’t step foot in a rehab facility, they don’t know what is available there. Right. And so I think that people need to work through the whole system. Like, that’s how we kind of know what actually works and what doesn’t. And, we need better collaboration instead of being siloed, right between our institutions and our specialties.” – 25G
**Facilitators to implementing the CGA**	
	“An efficient EMR [electronic medical record] has definitely helped me. I’ve used different ones. Having someone who would do parts of it like. Say if an OT [occupational therapist] like on the ACE [acute care of the elderly] team already did the cognitive assessment, then that saves me time. If you have a nurse who can help you with figuring out their medications, gathering history, social history, baseline functioning, I think those things would help.” – 01G “I think strong advocates on the units, whether they are charge nurses, whether they are allied health providers, whether we have some very strong-headed social workers, sometimes physio and occupational therapist, like from allied health, they’ll say, ‘Hey, listen, this person has Parkinson’s. Can you please get the geriatrician to come and assess this patient?’” – 02G “So at our hospital, the one thing that facilitates it is we have something called a delirium team. So a delirium team is, it’s essentially like an occupational therapist at each site. We have standard CAM [confusion assessment method] scores that are done on every patient by the nursing staff, not always accurate, but they’re done. And then if a patient has two consecutive positive CAM scores, the delirium team gets flagged. And their role is to, number one, like see the patient, assess for delirium, educate the staff on how to prevent and manage delirium from a non-pharmacologic perspective. But they also act as flags when they see a patient, given their experience with geriatrics, to say, this person should see a geriatrician.” – 04M “I think hiring more geriatricians, having more residents and fellows, um, on the service, having, um, a nurse practitioner as well, I think is probably also incredibly helpful.” – 12R “If we didn’t have such a health care shortage in general, like if I was thinking like what would be the ideal, I think this is what it should be. [Once] you get to a certain age, you get a referral [and] you keep your main physician, and… they are part of your team. I think that’s probably the best practice… like [a] best in class… model.” – 24C
**Barriers to implementing the CGA**	
	“Yes, I think I appreciate that geriatricians right now, we have a more consultative model, in that we do a great CGA, it’s detailed, it’s comprehensive, but we tend to leave the recommendations with the hopes that there’s other community partners who can help implement that. But I think the reality is we have to recognize that in the limitations of our system, you can’t expect that there will be someone there to implement those recommendations.” – 09M “I do wish that the outpatient clinic was a bit more willing to follow some patients more longitudinally over a year or two, for example. I find that like many other clinics, it tends to be a single consultation and then return to family doc, which may be appropriate, but, um, you know, I, when I see the patients for followup, often most of the recommendations have not really been carried out.” – 17R “[Without a team, ] I think you’ll have a lot of turnover [of healthcare providers], and I think you won’t have as much interest from people. New [geriatrician] grads don’t want to work at a place where they’re totally isolated. Gone are those days. I don’t even want to work in a place that I don’t have any other team support or other geriatricians as a new grad, right?” – 27M

### Theme 1: a broken healthcare system for older adults

Participants viewed geriatricians as advocates, educators and gatekeepers of the healthcare system for older adults. However, long wait times were consistently cited as a problem with accessing geriatric care in the outpatient setting. Patient participant 13 reflected “just in terms of getting… you know, an appointment took a long time… The whole process of getting [partner’s name] diagnosed, it took maybe two years… all together two years.” Participant 9, a health administrator, further explained the length of the geriatric assessment as an issue, “the assessment itself is so time consuming, you’re very limited in terms of the number of patients you can see. And so, because of that, there’s an extensive waitlist.”

However, another administrator, participant 19 disagreed with focusing on shortening the waitlist, attributing lack of integrated teams in the health system as the actual problem: “Geriatricians are trying to find a solution for our long wait lists. And if we try to do that, in exclusion of the system, we’re not ultimately helping the system, because it’s a broken system. So, we’ll cut the geriatric comprehensive geriatric assessment in half, we’ll tie ourselves into knots. I unfortunately, I don’t see that as the solution. So integrated teams providing population health, and working much more closely with each other actually talking to each other is what I see as the remedy for change.”

One geriatrician administrator (participant 9) reported a sense of personal guilt because the wait times were so long. They said, “for example, when you have a patient who sees you and they tell you, ‘Oh, we’re so excited to come see you, but it was like a year and a half wait time.’ And they tell you this. There is a little bit of a personal guilt there.” Along the same line, participant 19 added, “specialists are demoralized because they realize they cannot see their patients in a timely way.”

Acknowledging the shortage of geriatricians, several participants discussed the concept of “big G little g”. Participant 25 explained, “there’s big G as in us geriatricians, we’re providing the care for the most frail and medically complex patients. But there’s also little g, those other [care providers] who, we really need to try and educate about geriatric principles, so they could also support the older adult population.” Participant 2 thought that physicians “should all go through a robust geriatric rotation” during training to learn geriatric principles. At the hospital level, participant 20 wanted to “equip… the organisation and create translational knowledge…[that was] not restricted to internal medicine specialists” in order to “really disseminate small-g geriatrics and education across the organisation.” At a societal level, participant 27 said geriatric education should cover “not just all healthcare workers… [but also] the banker, the lawyer… grocery store owner.”

Most of the clinician and administrator participants indicated that hospital decision making was mainly driven by cost savings. Participant 6 stated, “[hospital administrators] perk up when you start talking about reduction in length of stay and admission rates. They don’t care about… the soft…indicators of good patient care. So I think we have to [show them] how we’re actually helping the hospital fiscally with CGAs.” Participant 8, a referring physician, similarly stated, “it seems like money is their language.” Participants mainly listed length of stay and (re)admission rates as the outcomes most important to hospital administrators, while a minority of participants listed mortality, complications (e.g. falls and delirium) and long-term care admissions as relevant outcomes. Participant 25 wanted outcomes like “patient satisfaction and…quality of life” to play a more important role in decision making, but acknowledged that this was unlikely. For geriatric services, participant 9 noted that “the problem, I think, with geriatrics is you don’t see the financial benefits of it very often.” Nearly all the clinician participants indicated a need to demonstrate the benefits of a geriatrician service to advocate for more funding and resources, in contrast to other clinical programs. The pressure to demonstrate a benefit of a CGA led participant 4 to ask “what’s the evidence for CCUs [coronary care units]? Like, there isn’t any, like none, right? Do they save lives? We don’t know, probably not. Why isn’t there a delirium unit? Why isn’t our [acute care of the elderly] unit taking up 200 beds in the hospital and not 26?”

Many participants described examples of ageism in the health system and in the larger society. Caregiver participant 18 stated, “you always hear the horror stories of caregivers in those settings [long term care] that don’t really care. And they don’t treat [older adults] like human beings, they just treat them like a number, and they have to be fed today, and they have to bath today.” Reflecting on their experience in the clinic setting, patient participant 22 stated that “you don’t want [the geriatric clinic] to be tucked away at the … last floor of the building… like I’m going to send you into a funeral parlour. It should look like the rest of the of the hospital, it should not be a downgrade.” Participant 22 also alluded to the concept of intergenerational contact (direct or indirect exposure of younger adults to older adults [43]), saying “I think that’s diminished greatly from my generation to my son’s generation to his son’s generation… I think that has diminished especially in the Anglo-Saxon population.” Upon discussing the lack of respect for older adults in day-to-day life, patient participant 21 said, “at one time, elders were honored, they were important. [in] a lot of societies… now, they just become old and useless.”

The scale of change needed in our health system to improve geriatric care was compared to the Krever commission [44] in haematology by participant 10, a referring physician. In the 1980s, many Canadians were harmed by tainted blood transfusions because of insufficient screening of blood products for HIV and hepatitis C. “We caused incredible harm with the way things were running… unavoidable rates of transmission. And it took a massive overhaul to wipe out the Red Cross blood services, and create the Canadian blood services. It took probably billions of dollars, but we are now the most revered blood conservation/transfusion organisation in the world.” Participant 10 added, “wouldn’t it be great if we could in geriatrics become leaders and get a lot of money to make a premiere [program] where people actually follow us.”

### Theme 2: varied perceptions of the impact of a CGA

Some participants reported that the benefits of a CGA were not well known by policymakers and even clinicians, which led to underutilization of this intervention. Participant 7, a health administrator, stated, “the benefits of the CGA are probably not very widely known. From a political point of view, for sure it’s not well known… I know a lot of family docs don’t even refer to geriatrics because they don’t really know what the point is because…sometimes, well, you can’t treat it [diseases related to aging].” Participant 10, a referring physician, recalled situations where patients were hesitant to see a geriatrician, stating “people don’t understand [a CGA]… even when I bring it up to patients to… see a geriatrician… they’re fearful of it.” Caregiver participant 15 recalled trying to access a geriatrician by “going through the family doctor, [but] they’ve never once said you should go to a geriatrician.” Patient participant 23 had a similar thought adding “I mean, does… every GP [general practitioner] know this service exists?” Other participants expressed more certainty about the benefits of the CGA. Participant 10, a referring physician, noted that “bounce back rates [will] go down by at least 20 to 30%” with a CGA. Other CGA benefits reported by participants included reducing polypharmacy, cognitive decline, functional decline, responsive behaviours and falls.

There were contrasting views about the effect of geriatrician involvement on length of stay in the acute care setting because of its potential effect on hospital resources. Participant 12, a referring physician, said that surgeons did not want their patients to be seen by geriatrics because “they probably worry that it lengthens their hospital stay – I’m just being honest.” Participant 6, a geriatrician administrator echoed that thought: “I can’t get the surgeons to appreciate what I can offer their patients.” Participant 7 observed that the perception was not limited to surgeons: “[length of stay is] usually priority one for the clinicians, the MRP [most responsible physician], whether it’s [an] internist or surgeon. If we’re going to do anything that potentially delays discharge, it’s not desirable.” Contrary to those views, participant 2 reported that a geriatrician-led CGA “definitely decreases length of stay.” Participant 4 cited data that complex patients on the geriatric unit at their hospital stayed “five days less in the hospital than a typical medicine patient.” Participant 8, a referring surgeon, stated “in my experience, I think length of stay is usually shorter [with geriatrician involvement] and discharge planning becomes much simpler.”

Out-of-pocket expenses was another perceived impact of a geriatrician-led CGA. Most of the patient and caregiver participants did not recall having to pay for anything out-of-pocket from the recommendations of a CGA. However, patient participant 13 recalled having to pay for a workbook ($75) for the Learning the Ropes program for mild cognitive impairment [45]. Participant 15, a caregiver, also noted that there was a cost for transportation to the clinic, for parking, and for time off from work because they had to attend the appointment. “And for me, it would be just like my time… off work (participant 15).”

### Theme 3: divergent views on healthcare setting for CGA

Participants reported that the geriatrician-led CGA was beneficial and acceptable in all healthcare settings. However, each participant was also asked which setting was most important for a geriatrician to staff. Patients and caregivers were generally not able to answer this question as they only experienced a CGA in one setting. Among the clinician and administrator participants, there were divergent views, especially between the acute care and community clinic settings. Rehabilitation was thought to be an important setting, but there was consensus that acute care or clinics should be staffed first. Some participants emphasized the need for geriatricians to be exposed to all of the settings to adequately understand the health system.

In support of a focus on acute care, referring surgeon participant 8 preferred geriatrician staffing in an acute care inpatient setting over an outpatient community setting because “very little is done of impact in the outpatient setting that doesn’t also centre around an inpatient admission.” Referring family physician participant 5 had similar views, stating that the preferred setting “should be absolutely in the hospital” because of the complexity of patients. Participant 5 added that “outpatient clinics… could be completed by somebody else who gets extra training like a family doctor or maybe a nurse practitioner.” Several participants said that the internal medicine expertise of a geriatrician is best used in the acute care setting, where other providers are less likely to have a similar complement of skills. Geriatrician administrator participant 6 said that “we can do the most as an inpatient consultation service,” but also stated that “I’ve had arguments with my colleagues about this because some of them feel very strongly we should just be an outpatient-based specialty.”

In support of community clinics as the preferred setting, some participants emphasized that preventative care can only be provided in the community setting, before a hospitalization occurs. Participant 20, a health administrator, stated that “the greatest value in terms of geriatric specialty knowledge and comprehensive geriatric assessment is probably in a setting that’s community-based and ambulatory, where you’re actually working on more preventative medicine.” Participant 4, another health administrator, also said the community setting is most important “because that is where you will pick up people at the earliest possible [time].” Geriatrician participant 11 said that it was challenging to be comprehensive in an acute care setting compared to a clinic setting where “we have dedicated time to speak with family members, the patient themselves when they’re well and [are] able to communicate to us their value [and] goals.” Participant 7, a health administrator, said that the community setting is where geriatricians “fill the biggest gap that other clinicians are not able to fill… particularly when it comes to dementia care and both diagnostically [and] behavioural issues.”

For the rehabilitation setting, participants had mixed opinions. Geriatrician participant 11 reflected that “the rehab setting is probably the better time to connect patients to outpatient services because in the acute care setting, when things are in flux, you might not necessarily know what their eventual functional outcomes are.” Participant 14, a health administrator, explained that the rehabilitation setting is more important because it offers a range of services including “a falls clinic or outpatient program, which supports family doctors, and then our inpatient setting, which also supports transition back to the community.” Participant 14 also noted that the rehabilitation setting was key to helping “older adults stay out of hospital… [which is an] important part of a geriatrician’s role.” Although participant 26, a health administrator, appreciated the role of geriatricians in a rehabilitation setting. This participant said he “would direct them to other [settings] instead” because there would be “a really big impact in [those] other [settings]” without geriatricians. Participant 9, another health administrator had a similar opinion, stating “if you have a good distribution [of geriatricians] in acute care and in the outpatient setting, it might not be necessary in the rehab setting.”

Several participants noted that working across different healthcare settings may be advantageous for clinicians. Working in a mix of acute care, rehabilitation, community clinics and long-term care may help clinicians better understand the resources available. Geriatrician participant 25 said, “I think that people need to work through the whole system… that’s how we know what actually works and what doesn’t. And, we need better collaboration instead of being siloed between our institutions and our specialties.” Participant 20, a health administrator, further suggested that experience in different settings can help identify the optimal location where a patient should be treated: “We’ve done this as well, a couple of times, people who come into the clinic, and we’re like, ‘Oh, you actually need an inpatient rehab stay.’ So we move them through the clinic into inpatient rehab, and then move them back out into our clinic or the day hospital.”

### Facilitators and barriers to implementing a geriatrician-led CGA

Facilitators to implementing a geriatrician-led CGA included (i) a good electronic patient record system, (ii) better integration with primary care (iii) a multidisciplinary team, and (iv) innovative ways to identify patients most in need of a CGA. Geriatrician participant 11 observed that “on the acute care side we have a functioning electronic medical record [system] and, on the rehab side, we have no electronic medical record. It really takes for whatever reason three hours to do a comprehensive geriatric assessment when there’s no medical record [system].” To achieve better integration with primary care, family physician participant 5 suggested that geriatricians “could even just see the patients from our clinic… because it keeps things local and intimate. We sometimes talk to [other] specialists while they’re there. Patient satisfaction is great.” Regarding a team’s role in facilitating a geriatrician-led CGA, participant 1 said “an OT [can do] the cognitive assessment… and a nurse… can help [figure] out their medications [and] gather history.” Multidisciplinary team members can also advocate for a CGA if they were aware of the benefits. Participant 2 recalled having “strong advocates on the units, whether they are charge nurses [or] allied health providers… they’ll say, ‘Hey, listen, this person has Parkinson’s [disease]. Can you please get the geriatrician to come and assess this patient?’” As an innovative way to find patients with delirium who could benefit from a geriatrician-led CGA, participant 4 had a team that included an occupational therapist who reviewed the chart documentation for positive confusion assessment method (CAM) scores. In addition to providing education and management recommendations, the occupational therapist also flagged “a patient, given their experience with geriatrics, to say, this person should see a geriatrician.”

Barriers to implementing a geriatrician-led CGA included (i) consultative model where recommendations are made but not implemented, (ii) limited team building training and (iii) lack of resources or administrative support. Participant 9, a health administrator, reflected on the current consultative model of geriatric medicine where recommendations are made for other providers to implement, but “you can’t expect that there will be someone there to implement those recommendations.” Participant 7, a health administrator, said that hospital funding for more geriatric services was a barrier. As an example, participant 7 said, “we can cut our six- to eight-month wait list down to a month… which would be ideal, but we would need probably two to three times as much space and resource.” Focusing on the teamwork needed to conduct a CGA, participant 19 noted that “everything we do in geriatrics is really reliant on teamwork. Yet, it shocks me at how little effort we put into teaching and training about teamwork… Because teams are like marriage, you got to work at it.”

### Trends by sex, gender and participant type

There did not appear to be any trends by sex and gender in the reporting of healthcare setting preferences, facilitators or barriers to conducting a CGA. Similar numbers of participants from represented sexes and genders reported the opinions above.

Contrasting views were shared between multiple participants (e.g., which setting was a priority for geriatricians). Although multiple clinician participants provided preferences for the priority setting, those who strongly preferred the community setting were mainly geriatrician health administrators. Patient and caregiver participants had similar views on obtaining a geriatric assessment, wait times, and ageism in society.

## Discussion

We conducted the first qualitative study to understand the acceptability and feasibility of a geriatrician-led CGA in different healthcare settings. There were conflicting views on whether an acute care or community clinic setting was most important for geriatricians to staff if there was a shortage of geriatricians. Geriatrician staffing in rehabilitation settings was valued, but some participants wanted acute care or community clinics to be staffed first.

Some participants prioritised the acute care setting for geriatrician staffing, which aligns with the results of our economic evaluation (under review) [9]. The internal medicine background of geriatricians is best suited for the care of complex older adults who are hospitalized. For example, geriatricians can provide care as the attending physician in an acute geriatric unit, where acute medically unwell older adults can receive multidisciplinary care. The acute geriatric unit has been demonstrated to improve functional status [46], reduce LTC admission [46], and reduce complications like delirium and falls [47]. Alternatively, geriatricians can provide consultative care across multiple hospital wards. This consultative model has the advantage of reaching more hospitalized older adults, but as some participants brought up, it is important for recommendations to be implemented by the primary care team. Inpatient geriatric consultation teams have demonstrated reduced mortality that is sustained up to 8 months after discharge [48]. Our qualitative data showed that a surgeon and a family physician participant both agreed that the acute care setting is most important for a geriatrician to staff, which aligns well with the available evidence.

Participants who preferred the outpatient community setting were mainly geriatrician health administrators. The primary reason was that a community-based CGA can prevent hospitalizations, which is supported by evidence from a 2022 Cochrane review (unplanned hospitalization relative risk 0.83) [49]. However, based on administrative data of Ontario older adults (age > 66 years) with high healthcare utilization (matched 1:3 for high- and low-cost users), 27.4% of the total cohort had an index hospitalization, but only 2.1% of the cohort received a geriatrician-led CGA at baseline [50]. This suggests that we need to increase the capacity for a geriatrician-led CGA by 13-fold to see all patients with a future hospitalization, assuming that we can perfectly identify patients who are going to be admitted to a hospital. Although staffing the community clinic setting is logical, there is insufficient geriatrician capacity to achieve the intended goal of reducing hospitalizations. Policymakers may consider training more geriatricians or determining whether other CGA providers can attain the effect of reducing hospitalization.

Although participants viewed staffing geriatricians in the rehabilitation setting to be important for helping with recovery and independent living, some administrators said that rehabilitation was relatively well supported if geriatricians were needed in other settings. This perspective does not align with findings from the economic evaluation, which found the combination of acute care and rehabilitation to be optimal. Furthermore, in a recent systematic review [51], geriatric rehabilitation was found to be effective in reducing mortality, long-term care admission, and improving function. Of the included geriatric rehabilitation trials, 69% included a geriatrician [51]. Despite this evidence, there are no data on how many rehabilitation patients are seen by a geriatrician in Ontario [52]. This highlights an important area of knowledge translation, so that evidence-based practices can be properly funded and implemented.

The CFIR was used to identify barriers and facilitators to implementing the geriatrician-led CGA in various healthcare settings [33]. Most facilitators and barriers described by the participants were rooted in cost and resource limitations. In the individuals domain, participants wanted stronger multidisciplinary teams and enough geriatricians to implement recommendations (implementation team members construct). In the inner setting domain (at the level of the organization/hospital), these factors included having team building training (relation connections and culture constructs), optimal clinic space (available resources construct), good electronic patient record system (information technology infrastructure construct), and programs to identify high risk patients (work infrastructure construct). In contrast to the tension for change in the inner setting, participants reflected on negative attitudes and values (ageism) in the outer setting (health system) that may be a barrier to improving the care of older adults. Future research can map the domains identified in the TDF and CFIR to the Capability, Opportunity, Motivation—Behaviour (COM-B) framework to create interventions for change [53].

There was near consensus from the clinician and administrator participants that hospital decision making was driven by cost savings. Several participants spoke about the need for individual geriatricians to demonstrate the benefits of a CGA locally at each hospital. While cancer care in Ontario has quality standards and best practices mandated throughout the province with dedicated funding [54], geriatric care has not received the same level of funding and coordination [55]. As an example, the lifetime risk of cancer for an adult in Ontario is 44.3% [56], the lifetime risk of dementia is comparable at 42.6% for a Canadian adult [57], which is just one of many geriatric syndromes that geriatricians manage. The significance of geriatric conditions on the health system warrants a concerted approach from the Ministry of Health to provide direct funding and create quality standards, similar to cancer care.

Limitations of this study included the use of an interviewer who was also a geriatrician (EW). Reflexivity was practiced throughout the study and interview technique was supervised by an experienced non-clinician qualitative expert (JS). There was a small number of types of referring physicians, but the group included participants from different fields (e.g. surgery, family medicine, medical subspecialties). The sample size was small but sufficient for qualitative studies [28]. Participants’ responses may have been influenced by the healthcare setting they worked in, thus favouring their own location of practice. We also did not achieve ideal representation of race and education categories in the study, which may limit the generalizability to underrepresented groups. There was an overrepresentation of white (67%) and east Asian (22%) participants compared to the Toronto area (42% and 13%, respectively) [25]. Race categories with fewer than five participants were not reported to preserve anonymity. Our participants had a higher proportion with postsecondary education (96%) compared with the Toronto population (62%), given the predominant inclusion of physicians and administrators. The interviews were conducted in English. Transcript coding was done by a single investigator (EW), but the analytic strategy was developed with a qualitative expert (JS) first.

There are several strengths to our study. We included a sample of participants with similar diversity of sex and gender as the Toronto area. The proportion of men + and women + categories in Toronto are 48% and 52%, respectively, which is the same as our participants [25]. We also applied an equity lens to attend to sex and gender differences in the findings and considered intersectionality. We used the theoretical domains framework [30] and the consolidated framework for implementation research 2.0 [33] to develop the interview guide, which helped to identify barriers and facilitators from an individual and organisational level. Key transcripts were reviewed by multiple analysts and direct quotations were used to support our results [41].

## Conclusions

Participants described a broken health care system for older adults and a varied perception of the impact of a CGA. They expressed good acceptability of staffing geriatricians in the acute care, community and rehabilitation settings. However, participants had different preferences of which setting to prioritise staffing if there was a shortage of geriatricians. The main barriers to implementing the geriatrician-led CGA related to cost and resource factors.

## Electronic supplementary material

Below is the link to the electronic supplementary material.

Supplementary Material 1: Appendix 1 (Interview guide)

## Data Availability

The datasets used and/or analysed during the current study are available from the corresponding author on reasonable request. Our research ethics board does not permit release of individual participant transcripts.

## Abbreviations

CGA: Comprehensive geriatric assessment
CFIR: Consolidated framework for implementation research
