# Supplementary material for: Perspectives on which health settings geriatricians should staff: a qualitative study of patients, care providers and health administrators
Source: BMC Geriatr. 2025 Jan 18;25:39. doi: 10.1186/s12877-025-05691-5 (PMC11748576; doi:10.1186/s12877-025-05691-5)
Supplement: Supplementary file 1 — Supplementary Material 1: Appendix 1 (Interview guide) [file 12877_2025_5691_MOESM1_ESM.docx]

**Interview guide for note taking Date of interview: ______________________________**

Indicate participant type: **Participant ID:** _________________________________

| **Patient/caregivers** | **Geriatricians and referring doctors** | **Health managers** | Domains from **TDF** and CFIR |
| --- | --- | --- | --- |
| Complete verbal consent form and demographic survey  Start recording / live transcription | | |  |
| **Preamble**: Thank you for participating in this interview. This discussion will be recorded for research purposes only and your personal information will not be shared. We hope to explore your experiences with a previous geriatric assessment. | **Preamble**: Thank you for participating in this interview. This discussion will be recorded for research purposes only and your personal information will not be shared. We hope to explore your experiences with (obtaining) a comprehensive geriatric assessment led by a geriatrician in the (acute care / outpatient clinic) setting. | **Preamble**: Thank you for participating in this interview. This discussion will be recorded for research purposes only and your personal information will not be shared. We hope to explore your experiences with managing comprehensive geriatric assessments led by a geriatrician. |  |
| 1. You (or your family member) may have seen a geriatric doctor recently. Why was the assessment done? What has your experience been to date with seeing doctors for your condition? | What is your experience with (conducting / obtaining) a geriatrician-led comprehensive geriatric assessment in (acute care / outpatient clinic) settings? Can you give me some examples of your experiences? (If clarification needed, indicate that this is a geriatric assessment conducted mainly or entirely by a geriatrician.) | What is your experience with managing geriatrician-led comprehensive geriatric assessment in (acute care / outpatient clinic) settings? Can you give me some examples of your experiences? (If clarification needed, indicate that this is a geriatric assessment conducted mainly or entirely by a geriatrician.) | **Knowledge**  Innovation (cost, evidence base, relative advantage, design, adaptability, complexity)  Individuals (leaders, team members, deliverers, recipients)  Intervention characteristics (need, motivation)  Implementation process (assessing needs, engaging deliverer/recipient, assessing context) |
| Were there any things you had to pay for in order to follow the recommendations of the geriatrics assessment?  Was there any cost not covered by the hospital? | **For referring physicians**: Why do you refer patients for a CGA? What are the differences when a geriatrician leads a CGA? (If asked, can bring up types of recommendations, issue identification, satisfaction)  **For geriatricians**: Which patients should get a CGA in the hospital setting? (e.g. frailty, disease, med/surg) Why | What issues can a geriatrician-led CGA address?  How can an organization adapt if more geriatrician-led CGAs are required than what is currently available? |  |
| 1. **For patients/caregivers:** In your opinion, what kind of skills should a geriatric doctor have when looking after patients admitted to the hospital (or in a clinic)?   **For physicians/managers:** In your opinion, what sort of skills (or competence) should a geriatrician have in acute care (or community) setting? (low priority)   Optional: Do geriatricians have those capabilities, scope or power? Please explain. (low priority) | | | **Skills**  **Beliefs about capabilities**  Intervention characteristics (capability, opportunity) |
| 1. **For patient/caregivers:** What kind of role should a geriatric doctor have in the health system? What else can a geriatric doctor do other than to provide care? (What kind of difference can a geriatrician make in a hospital?)  **For physicians/managers:** If geriatricians can only be focused in one setting (e.g. acute care, outpatient clinics, rehab, outreach or LTC), which setting should that be? And why? | | | **Role and identity**  Individuals (deliverers, leaders, facilitators, team members) |
|  | Do other professionals carry out a CGA? If so, who? (low priority)  How does a geriatrician provide leadership roles for your facility if they currently do? What would that look like? (low priority) | |  |
| 1. What would have happened if you did not see a geriatrician? If the service was not available? | What are the benefits of seeing all frail older adults in hospital?  What are the consequences if we do not see every frail older adult in hospital?  How would you feel if we concentrated all geriatricians in the acute care setting (e.g. ACE, inpatient consults)? Why so?  For community participants: How can we also staff geriatricians in outpatient clinic settings? | | **Optimism; beliefs about consequences; emotion**  Inner setting (structural, culture, tension for change, resources) |
| 1. **For patient/families:** In your opinion, what is needed to run a program or clinic for older adults? Examples can be things like nurses, other specialists, tools, handouts.  **For physician/managers:** In your opinion, what types of resources would be needed to conduct a geriatrician-led CGA? (if asked, may give examples like clinical environment, personnel, equipment, tools, supplies, other resources) | | | **Environmental context & resources**  Inner setting (relative priorities, structural, culture, tension for change, incentive systems, mission alignment, resources)  Implementation process (assessing needs, engaging deliverer/recipient, assessing context, planning, doing, reflecting/evaluating, adapting) |
| Were there obstacles in getting a geriatric assessment? If so, what were they?  Were there things or people who helped you get a geriatric assessment? If so, what or who were they? | *Barriers*: What things have interfered with implementing a geriatrician-led CGA for all frail older adults in acute care? (OK to give examples when prompted)  *Facilitators*: What things have helped to implement a geriatrician-led CGA for all frail older adults in acute care? | |  |
| 1. What incentive (or motivation?) can we give hospitals to provide more geriatrician-led CGAs? How so? (Optional for patients) | | | **Reinforcement**  **Intentions; goals**  Outer setting (external pressure, local attitudes, local conditions, financing, partnerships and connections)  Inner setting (structural, culture, tension for change, incentive systems, mission alignment, resources) |
|  | Does your hospital have any plans to do this?  What target percentage of frail older hospitalized adults should get a geriatrician-led CGA?  How would increasing geriatrician-led CGAs align with the mission of your program/hospital? | |  |
| 1. What types of external factors (factors the hospital cannot control) would influence hospitals to change the current number of CGAs provided? (e.g., media, political thinking) (Optional for patients)   Do you think that government policies and regulations are supportive of increasing geriatrician-led CGAs? How so? | | | **Social influences**  Outer setting (external pressure, local attitudes, policies/laws) |
| **Closing remarks**: Is there anything that you think I missed asking about today? Or anything else you would like to say about this topic? Thank you for participating. | | |  |

Gift card of choice:  Amazon.ca  Tim Hortons  President Choice  Visa/Mastercard prepaid

Digital card  Physical card
